# Left ventricular shape variation in patients with aortic coarctation pre- and post-stent implantation

**DOI:** 10.1186/1532-429X-18-S1-P186

**Published:** 2016-01-27

**Authors:** AK Ortiz, Avan Suinesiaputra, Alistair Young, Genevieve E Farrar, Beth F Printz, Jeff Omens, Andrew D McCulloch, James C Perry, Sanjeet Hegde

**Affiliations:** 1 University of California, San Diego, School of Medicine, La Jolla, CA USA; 2 Department of Bioengineering, University of California, San Diego, La Jolla, CA USA; 3 Department of Anatomy with Radiology, University of Auckland, New Zealand, Auckland, New Zealand; 4 Department of Medicine, University of California, San Diego, La Jolla CA USA; 5 Division of Cardiology, Rady Children's Hospital, San Diego, CA USA; 6 Department of Pediatrics, University of California, San Diego, La Jolla, CA USA

## Background

Coarctation of the aorta (CoA) affects about 4 out of every 10,000 babies born and requires lifelong follow-up. Even after successful early repair, late cardiovascular (CV) complications arise. In adult patients, left ventricular remodeling (LVR) patterns can predict CV risk. However, few studies on LVR of CoA patients exist due to lack of detailed geometric models. The Cardiac Atlas Project (CAP), a worldwide consortium for pooling standardized analyses of cardiac images for mapping heart shape and motion, provides a unique opportunity to study CoA cardiac remodeling. This study compares LV shape in a sample of CoA patients pre- and post-stent to normal patients from the CAP with the goal of developing model-based predictions of LVR to guide clinical management.

## Methods

Cardiac magnetic resonance images (CMRI) and clinical data of five patients (Ages: 8-24 yrs.) with native and re-coarctation of the aorta were collected with IRB approval. All study patients were potential candidates for stent implantation. CMRI 1-2yrs. post-stent are currently available for four patients.

Patient-specific geometric models were built from CMRI using finite element guide-point modeling software (CIM, University of Auckland, New Zealand). Shape modes at end-systole (ES) and end-diastole (ED) were derived using principal component analysis (PCA) of a population (n = 1991) of asymptomatic volunteers from the CAP. PC scores describing the deviation of each CoA model from the asymptomatic PCA model distribution at ED and ES for various shape modes were calculated.

## Results

**Figure 1:**
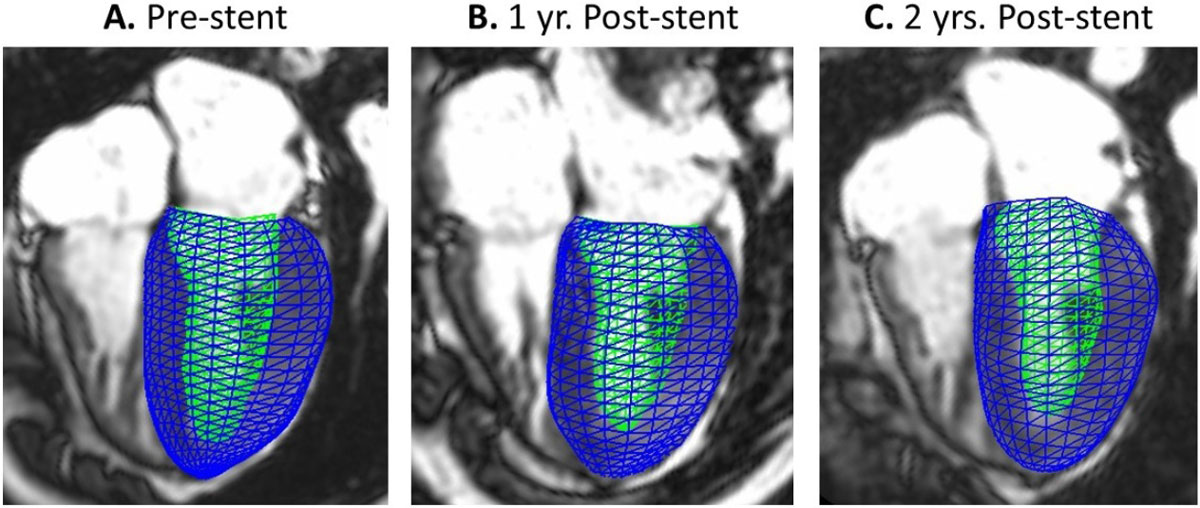
**Finite element meshes of representative geometric models pre- and post-stent implantation for a single patient at end systole**.

Figure 1 shows representative geometric models for a single patient and Table 1 reports PC scores for all patients. In order, PC modes (M1-M4) represent variance in: 1) LV size, 2) sphericity and wall thickness, 3) mitral valve orientation (MVO), and 4) wall thickness and MVO. Most PC scores are <2.0, indicating model shapes are within two standard deviations of the control population. At ED and ES pre-stent, the largest absolute PC score for most patients is for M4, which predominantly represents wall thickness and MVO. Generally, as the value for M4 post-stent decreases, the mode with the largest absolute PC changes for a particular patient even at one year post-stent. This is consistent with recent literature showing that short-term LVR may occur in CoA patients post-intervention.

**Table 1:** Standardized Principle Component (PC) Scores at End Systole and End Diastole

PC	% Var	Shape Characteristic	Patient 1			Patient 2			Patient 3			Patient 4			Patient 5		
			Pre	1 yr. Post	2 yrs. Post	Pre	1 yr. Post	2 yrs. Post	Pre	1 yr. Post	2 yrs. Post	Pre	1 yr. Post	2 yrs. Post	Pre	1 yr. Post	2 yrs. Post
End Systole																	
M1	43.1	LV Size	0.3	0.6	0.3	-0.1	-0.1	-0.4	**1.5**	-	-	1.3	**1.3**	-	0.8	-0.6	-
M2	11.0	Sphericity, Wall Thickness	-1.3	**-1.3**	0.1	**1.3**	**1.7**	1.3	-0.9	-	-	1.1	0.4	-	**-2.3**	0.9	-
M3	7.2	Mitral Valve Orientation	-0.7	0.7	0.3	0.5	0.6	**1.5**	-0.1	-	-	-0.9	-0.6	-	1.2	0.5	-
M4	5.0	Wall Thickness, Mitral Valve Orientation	**2.0**	0.7	**-1.2**	**-1.3**	-0.6	-0.9	-0.7	-	-	**-1.9**	-0.9	-	1.3	**-1.4**	-
End Diastole																	
M1	44.5	LV Size	0.0	0.1	0.3	-0.3	**-0.9**	-1.1	1.6	-	-	1.5	0.6	-	0.6	-0.3	-
M2	10.6	Sphericity, Wall Thickness	0.4	-0.3	0.2	-0.2	-0.8	-1.3	-0.3	-	-	1.2	0.3	-	**-1.2**	-0.4	-
M3	9.2	Mitral Valve Orientation	-0.1	**0.5**	**0.9**	-0.9	-0.3	-0.2	0.0	-	-	-0.4	**1.1**	-	0.5	**0.7**	-
M4	6.8	Wall Thickness, Mitral Valve Orientation	**1.1**	**0.5**	0.7	**1.5**	-0.3	**-1.5**	**1.9**	-	-	**1.6**	0.1	-	0.9	0.3	-

The four PC modes (M1-M4) represent the highest percentage of shape variance (% Var), with a total of 66% at ES and 71% at ED, within the asymptomatic population as compared to other shape modes derived from PCA (not shown). The shape characteristic represented by each mode is noted. PC with the largest absolute score is highlighted in bold for each model. Patient 3 has not undergone stent implantation. Patients 4 and 5 have not undergone 2-year follow-up CMR. Corresponding models and PC scores for Patients 3-5 are not available.

## Conclusions

Preliminary results with a small sample size comparing LV geometry of CoA patients against an atlas-based population of controls show LV shape characteristics that are similar to the normal LV. Notable characteristics post-stent include change in wall thickness and mitral valve orientation which reflect remodeling of the ventricle. Detailed quantification of cardiac shape can improve our understanding of the adverse effects of remodeling and, in combination with future patient-specific biomechanics models, advance the potential for early recognition of remodeling patterns to guide timely clinical management.

